# Clinical and Biometric Assessment of a Hyaluronic Acid‐Based Skin Booster for Face, Neck and Décolleté Rejuvenation: A Prospective Study

**DOI:** 10.1111/jocd.70547

**Published:** 2025-11-16

**Authors:** A. Pino, J. Torrecilla, J. M. Alonso, L. Tovito, R. Pérez

**Affiliations:** ^1^ i+Med S. Coop. Vitoria Spain; ^2^ Complutense Medical Centre (Virtus Group) Madrid Spain

**Keywords:** aesthetic medicine, biometric assessment, hyaluronic acid, rejuvenation, skinbooster

## Abstract

**Background:**

Hyaluronic acid based skinboosters present a low rheological profile that allows for even distribution throughout the dermis and focuses on improving skin's overall quality. Scientific efforts are ongoing with the aim of developing optimal skinboosters that meet specific clinical requirements and satisfactory overall results.

**Objective:**

Assess clinically the efficacy and safety of a newly developed skinbooster known as BtHLINE for face, neck and décolleté rejuvenation.

**Materials and Methods:**

In this prospective and single‐centre clinical trial, 81 volunteers with signs of cutaneous aging received three intradermal injections of BtHLINE every 3 weeks and were followed up for 15 days, 3 months and 6 months. Efficacy evaluation included the Multifactorial Aesthetic Scale (MAS), the Global Aesthetic Improvement Scale (GAIS) and the Likert's patient satisfaction questionnaire. Biometric parameters were also assessed over the facial area using specific aesthetic instrumentation such as Visioscan, Cutometer, Moisturemap and Visioface tools. Adverse events related to the treatment were registered throughout the study.

**Results:**

MAS score reached a statistically significant improvement at each time point for every treated area. Accordingly, GAIS and patient satisfaction surveys showed a clinically noticeable percentage of responders at the end of the follow‐up. The biometric analysis revealed that BtHLINE promoted a rapid and significant improvement that was maintained during the follow‐up period in terms of cutaneous uniformity, viscoelasticity, flexibility, firmness, water content, hydration, wrinkle depth, surface irregularities, scaling and smoothness. Adverse events were slight and considered as usual among skinboosters and were resolved within a few days.

**Conclusion:**

This study demonstrates that BtHLINE is safe and effective for the promotion of skin rejuvenation.

## 1 Introduction

As external appearance plays a crucial role in social interactions and the skin is the most visible organ, cutaneous aging becomes a significant concern. Skin aging occurs due to cellular and intercellular matrix degradation, decreased vascularization, dysfunction of skin appendages, fat atrophy and reduced cellular function. These processes are primarily driven by genetics through a set of molecular and cellular mechanisms [1]. Other contributing factors include the exposome that directly affects the cutaneous tissue such as solar damage, dermatological and systemic diseases, hormonal status, nutrition and toxic habits. As a result, the most evident signs of aging appear, including wrinkles, dyschromia, and ptosis, along with less noticeable changes in texture, smoothness, tone, hydration, elasticity and radiance. All of these changes collectively determine the overall appearance of the skin [2]. The pursuit of maintaining a youthful and healthy look is nowadays considered acceptable or even necessary in some social and professional fields. Thus, the growing aesthetic awareness has led to a rising interest in developing new products that help improve the appearance of skin.

In this regard, a wide range of therapeutic options is available to address the main signs of skin aging. Aesthetic treatments using injectables have become increasingly popular for those patients seeking a fast and non‐surgical rejuvenation. Injectable skinboosters are non‐permanent products specifically designed to improve cutaneous quality. Unlike semi‐permanent options like dermal fillers which seek to reshape and volumize the contour by structural enhancement, skinboosters focus on improving skin's overall appearance and target cutaneous hydration, elasticity, smoothness and radiance [3, 4, 5]. These products usually present a more fluid and lower rheological profile compared to conventional fillers. They display reduced viscous/elastic modulus which allows an even distribution throughout the dermis once injected on a multiple session basis. Hence, skinboosters are not intended to define contours or restore deep wrinkles or folds, but to promote cutaneous condition by superficial texture and tone recovery which finally improves overall skin quality in a more natural and gradual way [6].

Hyaluronic acid (HA) is an endogenous glycosaminoglycan that is commonly used as the main component of skinboosters. This natural biopolymer is found in the extracellular matrix and is one of the main components of the dermal connective tissue that helps maintain skin structure and function [7]. Microinjections of linear HA may promote the absorption of large amounts of water attracting and retaining moisture in the dermis. Furthermore, HA forms complexes with collagen and elastin which support skin structure and cellular metabolism. However, the aging process leads to a decline in the skin's natural HA turnover and both the concentration and the molecular weight of the polymer chain are reduced [8]. Therefore, the precise injection of small quantities of linear HA into the superficial and mid dermal layers helps the restoration of the optimal extracellular environment that is needed for surrounding biological components.

Remarkable scientific efforts are currently ongoing with the aim of developing optimal skinboosters that meet specific rheological and clinical requirements. Accordingly, every innovation should be assessed through biometric tools and validated scales in the context of a clinical trial to demonstrate the final clinical performance in terms of aesthetic improvement. In this respect, the aim of this study is to assess the efficacy and safety of a new skinbooster known as BtHLINE in the management of skin rejuvenation.

## 2 Materials and Methods

### 2.1 Study Design

This study was designed as a prospective clinical trial and was carried out in a single centre (Complutense Medical Centre‐Virtus Group, Madrid, Spain). This trial was reported following the Strengthening the Reporting of Observational Studies in Epidemiology (STROBE) Statement [9]. The study protocol (code BtHLINE‐PIC01‐2021) as well as the subject information sheet and informed consent form, were reviewed and approved on 30 March 2022 by the ethics committee of the University Hospital Principe de Asturias (code PIPS01/2022, Madrid, Spain) in accordance with the international ethical standards from the revised World Medical Association Declaration of Helsinki amended in 2013. Participants provided a specific informed consent for publication of the photographs taken during the study. This study was conducted following the guidelines established in UNE‐EN ISO 14155:2021 (Clinical research on medical devices for humans. Good clinical practices) and CPMP/ICH‐GCP‐E6/135/95 (Guidance on Good Clinical Practice of the European Medicines Agency).

### 2.2 Subject Selection

Subjects provided written informed consent before entry into the study and were recruited from April 2022 and followed till December 2022. A preliminary assessment of each subject was carried out by an aesthetic clinician at the baseline visit and the medical history was completed. Volunteers were included in the study if they met all the inclusion/exclusion criteria shown in Table 1. Subjects received three intradermal injections of BtHLINE every 3 weeks and after the last injection returned for follow‐up at 15 days, 3 months and 6 months. These time points were selected based on the deep knowledge of the optimized polymer structure of the product and its theoretical clinical performance following expert opinion in the field. The statistical data processing of the clinical results was performed by researchers not involved in the clinical intervention; hence no unblinding occurred during the data analysis.

**TABLE 1:** Inclusion and exclusion criteria for study participation.

Inclusion criteria	Exclusion criteria
– Men or women between 30 and 70 years of age. – Subjects that present facial aging symptoms including decreased radiance, hydration or firmness. – Willingness and ability to complete questionnaires and understand study instructions. – Signed informed consent specific to the study.	– Pregnant or breastfeeding women. – Allergy or sensitivity to hyaluronic acid or any other ingredient of the product under study. – Have undergone any rejuvenation or wrinkle correction procedure in the 6 months prior to the study entry or during the study such as radiofrequency, electrotherapy, botulinum toxin, tensor threads, platelet rich plasma or laser techniques. – Autoimmune or inflammatory diseases that comprises the condition of the skin. – Disease or active infection in the area to be treated. – Lymphatic and/or vascular pathology. – Previous facial surgery. – Blood coagulation disorders or in treatment with anticoagulants. – Disproportionate expectations regarding the clinical performance of the product.

### 2.3 Intervention

BtHLINE is a hyaluronic acid‐based skinbooster with CE approval which is classified as a Class III medical device and has a sodium hyaluronate concentration of 1.4% (14 mg/mL). All subjects in the study were scheduled for the baseline visit and received three intradermal injections of BtHLINE every 3 weeks. One milliliter of BtHLINE was applied over the facial (forehead, perioral/periocular areas and cheeks), neck (venus rings) and décolleté (fine wrinkles) areas at the level of the middle and superficial dermis using 30G needles and a linear retrograde injection technique. Lidocaine cream was used as topical anesthesia before each intervention.

### 2.4 Outcome Measures

#### 2.4.1 Efficacy Assessment

The clinical efficacy quantitative assessment was performed by one blinded and trained technician. This technician was not involved in the intervention procedure (product injection) or in the statistical data processing. Primary and secondary outcomes were selected by experienced investigators regarding clinical improvement objectivation as a common tool in daily aesthetic procedures.

The primary efficacy outcome was the clinical improvement observed by an aesthetic specialist using the Multifactorial Aesthetic Scale (MAS) (Table S1). This scale includes five dimensions (radiance, hydration, firmness, fine lines and overall rejuvenation) associated with a five‐point score (0‐very poor status, 1‐poor status, 2‐normal status, 3‐good status, 4‐very good status). The MAS was assessed at baseline and during the follow‐up visits (that were scheduled after the last injection session) at 15 days, 3 months and 6 months. Independent MAS scores were determined for each treated area (face, neck and decolleté).

The secondary efficacy outcomes included the clinical improvement as assessed by the clinicians using the five‐point Global Aesthetic Improvement Scale (GAIS) (subject percentage reporting “slight improvement”, “improvement” or “important improvement” was calculated as responders). The five‐point Likert's patient satisfaction questionnaire completed by the volunteers was also reported (subject percentage reporting “slightly satisfied”, “satisfied” or “very satisfied” was calculated as responders). These questionnaires were evaluated at baseline and during the follow‐up visits (that were scheduled after the last injection session) at 15 days, 3 months and 6 months. Independent GAIS and satisfaction scores were determined for each treated area (face, neck and decolleté).

In addition, several biometric outcomes were studied quantitatively using specific aesthetic instrumentation (Courage Khazaka Electronics, Cologne, Germany). The Visioscan analysis probe comprises a specific high‐resolution UV‐A light video camera, designed to study the epidermal topography. The integrated SELS technology (Surface Evaluation of the Living Skin), which analyses different gray scale distributions of images, allows the quantitative calculation of clinical parameters such as skin uniformity, roughness, scaling, wrinkle depth, surface irregularities or smoothness [10]. Cutometer analysis probe is a widely used dermatological tool based on the cutaneous suction and relaxation method. It creates a local negative pressure that draws the skin surface into an optical system where a light source connected to two projecting prisms, quantitatively determines the cutaneous behavior in terms of flexibility, firmness and viscoelasticity [11]. Moisturemap analysis probe generates topographic images by capacitance that provide information about the distribution of hydration and water content near the skin surface [12]. Finally, the Visioface is a digital camera that employs a 200 LED source of backlight to obtain standardized and high‐resolution images of the entire facial area which may be evaluated for wrinkles, pores, spots or UV spots [13]. Biometric assessment was conducted over the same areas throughout the study in a temperature and humidity‐controlled atmosphere. Biometric outcomes were assessed at baseline, at each treatment visit and during the follow‐up visits (that were scheduled after the last injection session) at 15 days, 3 months and 6 months. Biometric analysis was performed over the facial area by taking measurements at the forehead, perioral/periocular areas, and cheeks.

#### 2.4.2 Safety Assessment

The nature, onset, duration, severity and outcomes of all adverse events, as well as any association of an adverse event related to the study treatment according to specialist criteria, were assessed and registered at each visit. To evaluate the safety profile of the treatment all complications and adverse events were recorded with an accountability scale.

### 2.5 Statistical Analysis

Initially, a descriptive analysis of the sample was performed considering the demographics and baseline clinical variables of patients. Quantitative variables were determined by the mean, standard deviation and range, and for qualitative variables, a frequencies analysis was conducted. Multiple measurements collected over time (consecutive measurements for each volunteer), and therefore correlated with each other, were considered by including random events at the volunteer level. Such an approach allows the intercept to vary randomly between volunteers. Linear mixed‐event models (LMM) were fitted to assess treatment efficacy over time. A two‐tailed *p*‐value for repeated means was calculated. For clinical outcomes a descriptive analysis was performed using frequency distributions in the case of qualitative parameters and mean and standard deviation (or median and interquartile range) for quantitative variables. Normality, homogeneity of variances and homoscedasticity analysis were performed. Depending on the conditions met, a nonparametric analysis (Kruskal Wallis) or a parametric test (ANOVA) was performed. The significance level was set at 0.05 and confidence intervals were calculated at 95%. The SPSS statistical package version 29.0 and R‐Studio version 4.3.2 were used for the statistical analysis.

## 3 Results

Subject demographics at baseline are shown in Table 2. A total of 81 patients were included in the study and completed the follow‐up. The average age was 48.27 ± 8.61 years and the female/male ratio was 86.42%/13.58%. A total of 34, 24 and 23 subjects were treated with BtHLINE over the face, neck and décolleté areas respectively and the injected volume in each area was 1 mL at each treatment visit for a total of 3 mL.

**TABLE 2:** Baseline demographics of subjects included in the study.

	Face	Neck	Décolleté
Sample size (*N*)	34	24	23
Age (years)	47.58 ± 8.36	48.58 ± 9.52	48.87 ± 8.31
Gender (%): female/male	73.5/26.5	91.7/8.3	95.7/4.3

Patients did not refer remarkable procedural discomfort. Safety data regarding adverse events are summarized in Table 3. During the study, no adverse events were observed apart from transient pain, slight swelling/oedema, mild hematoma or slight itching and bleeding in some of the volunteers. These expected technique‐dependent events were mild to moderate and were mostly resolved within a few days following the injection procedure. Hence, BtHLINE could be considered a safe treatment.

**TABLE 3:** Adverse events (1°injection, 2°injection, 3°injection, 15 days, 3 months, and 6 months).

	Face	Neck	Décolleté
Pain: event % 1°inj/2°inj/3°inj/15d/3 m/6 m	41%/32%/24%/0%/0%/0%	17%/13%/8%/0%/0%/0%	35%/22%/7%/0%/0%/0%
Swelling/oedema: event % 1°inj/2°inj/3°inj/15d/3 m/6 m	29%/15%/12%/0%/0%/0%	29%/4%/8%/0%/0%/0%	39%/0%/4%/0%/0%/0%
Hematoma: event % 1°inj/2°inj/3°inj/15d/3 m/6 m	15%/0%/0%/0%/0%/0%	4%/0%/0%/0%/0%/0%	4%/0%/0%/0%/0%/0%
Itching: event % 1°inj/2°inj/3°inj/15d/3 m/6 m	6%/3%/9%/0%/0%/0%	4%/4%/4%/0%/0%/0%	4%/13%/7%/0%/0%/0%
Bleeding: event % 1°inj/2°inj/3°inj/15d/3 m/6 m	9%/9%/9%/0%/0%/0%	0%/0%/0%/0%/0%/0%	0%/0%/0%/0%/0%/0%

**FIGURE 1:**
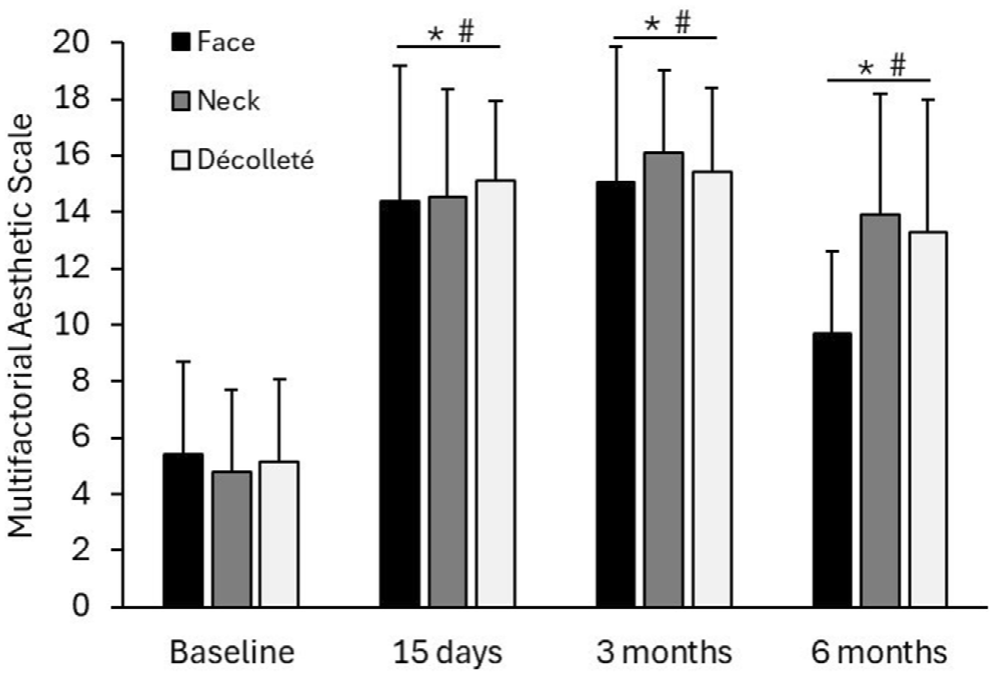
Clinical improvement for BtHLINE treated areas assessed by the Multifactorial Aesthetic Scale. An increase in the score is associated with a clinical improvement. *: Statistically significant differences compared to baseline (*p* ≤ 0.05). #: ≥ 5 point improvement in the score.

**FIGURE 2:**
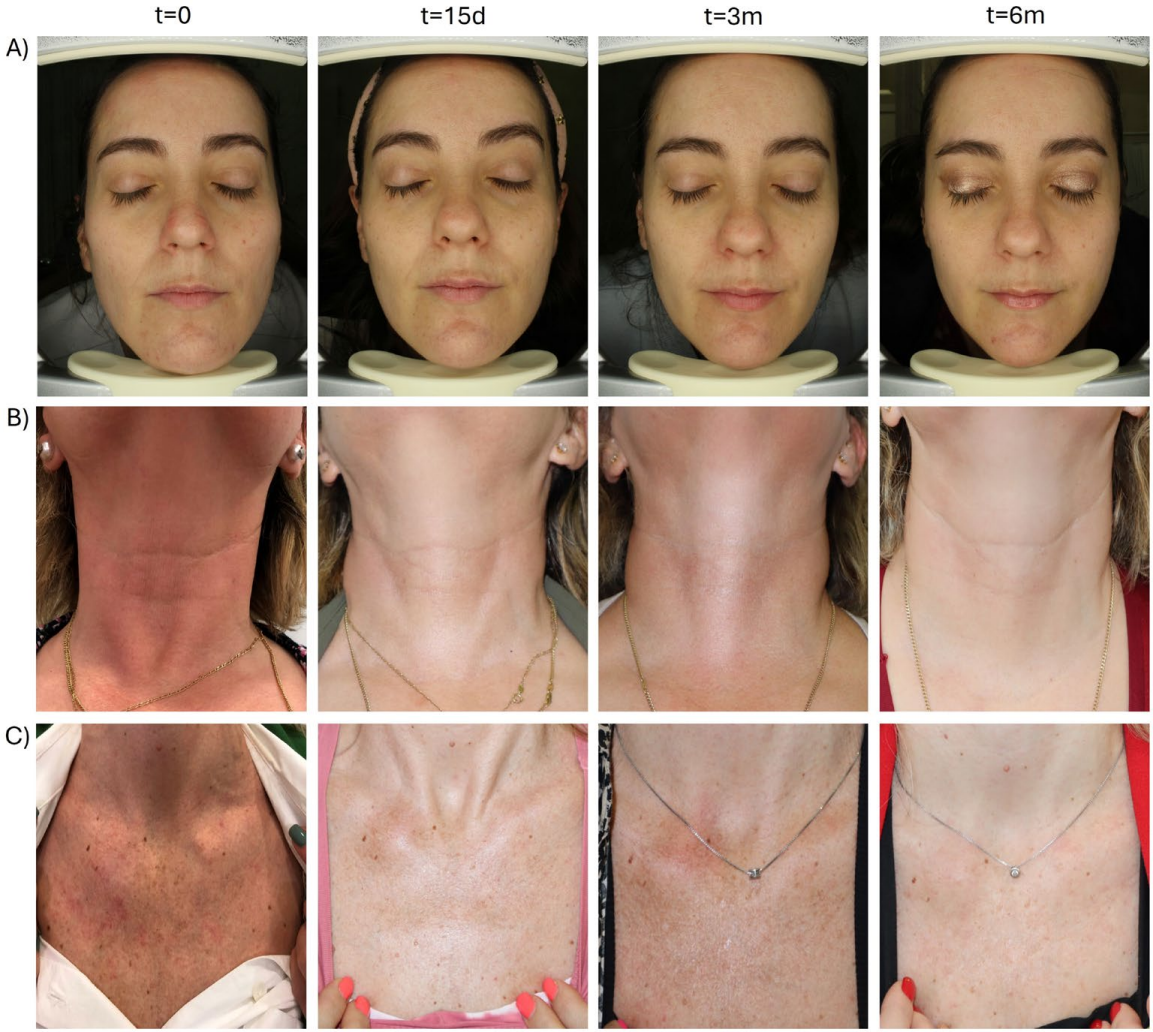
Standardized photographs illustrating the clinical evolution of three volunteers at different time points. (A) Evolution of the facial area. (B) Evolution of the neck area. (C) Evolution of the décolleté area.

**FIGURE 3:**
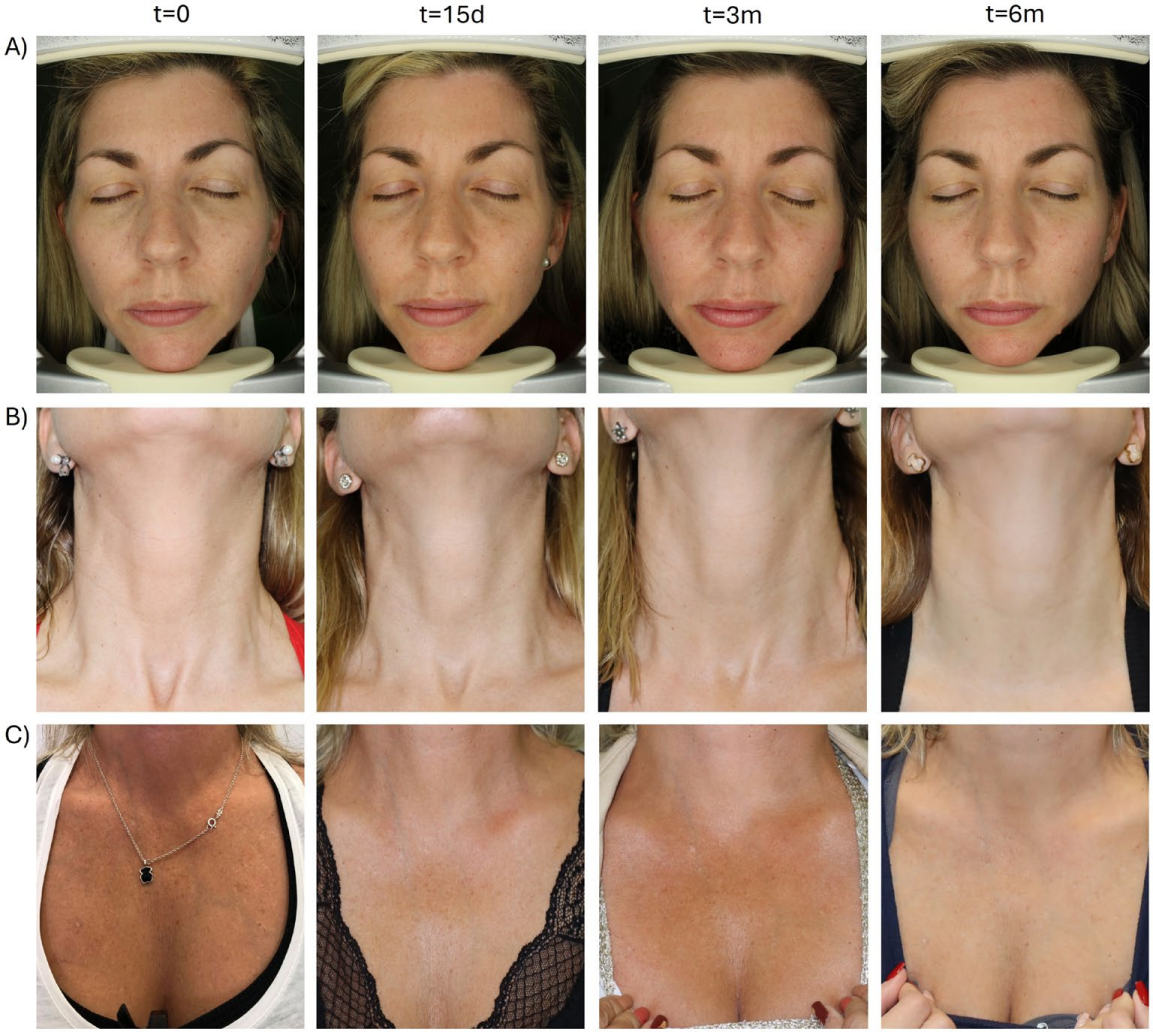
Standardized photographs illustrating the clinical evolution of three volunteers at different time points. (A) Evolution of the facial area. (B) Evolution of the neck area. (C) Evolution of the décolleté area.

The clinical improvement was assessed by the Multifactorial Aesthetic Scale completed by clinicians. Baseline MAS score indicated that included participants formed a homogeneous population regarding the overall aging status before the application of the treatment. A statistically significant improvement could be observed for all the treated areas when compared to baseline status (*p* ≤ 0.05). Early and clinically relevant results were noticeable after 15 days and were maintained for 3 months and 6 months (Figure 1). Furthermore, when analyzing the pre‐ and post‐treatment status, specialists reported a global aesthetic improvement in over 85%, 85% and 60% of the volunteers at 15 days, 3 months and 6 months respectively (GAIS scale). Results of the subjective satisfaction referred by participants (Likert's questionnaire) showed a steady increase in responder percentage that reached 91%, 88% and 86% at the end of the follow up for face, neck and décolleté areas respectively. Table 4 summarizes GAIS and patient satisfaction results. Figure 2 and Figure 3 show standardized photographs illustrating the clinical evolution of volunteers at different time points.

**TABLE 4:** GAIS and patient satisfaction results of the treated areas throughout the study.

		15 days	3 months	6 months
GAIS (responder %)	Face	88%	88%	61%
	Neck	88%	88%	88%
	Décolleté	92%	92%	77%
Patient satisfaction (responder %)	Face	38%	38%	91%
	Neck	40%	44%	88%
	Décolleté	50%	54%	86%

**FIGURE 4:**
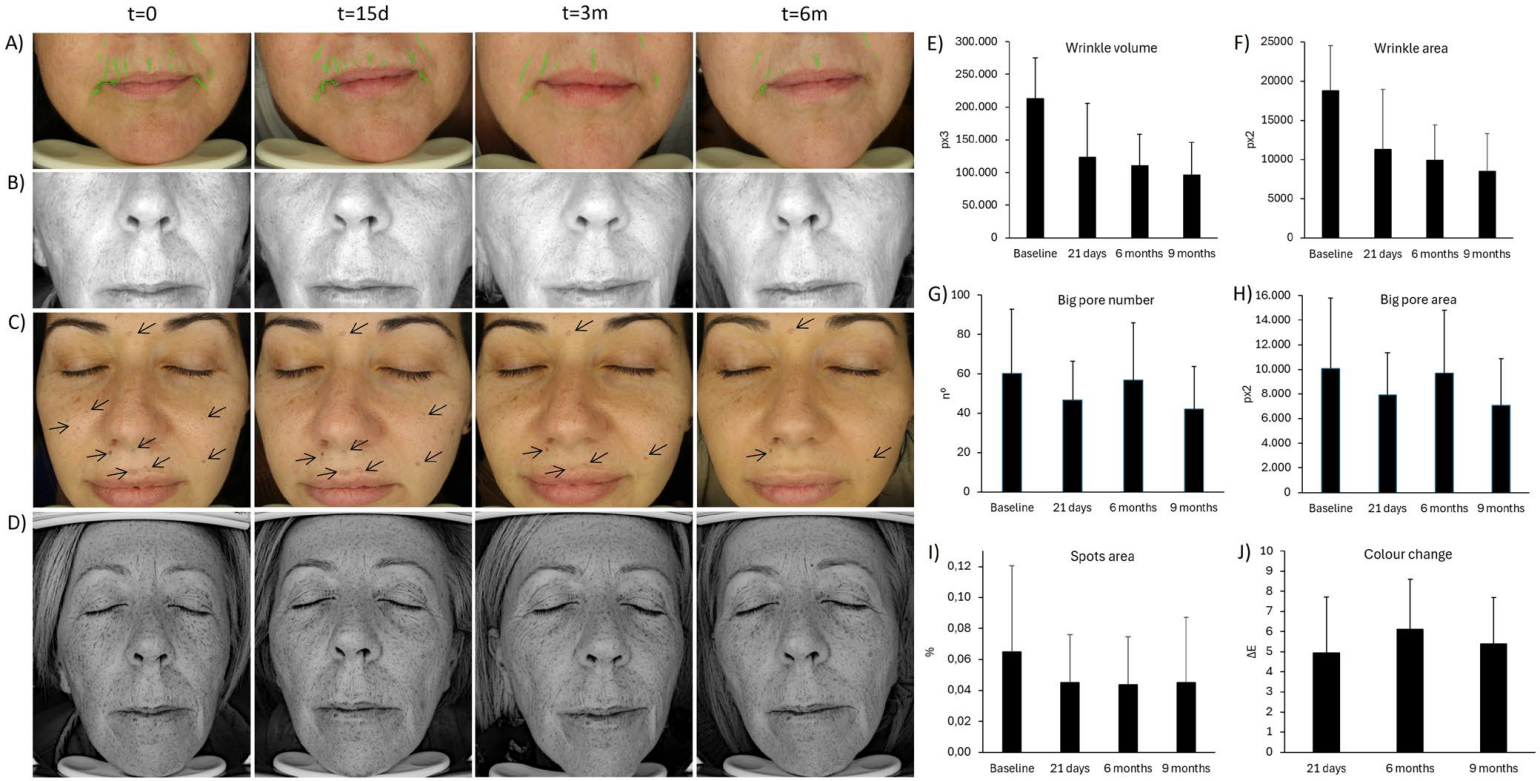
Representative assessment using the Visioface camera. (A) Images for wrinkle analysis. (B) Images for pore analysis. (C) Images for spots analysis. (D) Images of UV spots. (E) Wrinkle volume. (F) Wrinkle area. (G) Big pore number. (H) Big pore area. (I) Spots area. (J) Color change. Data assessment comprises a representative analysis of three subjects.

**FIGURE 5:**
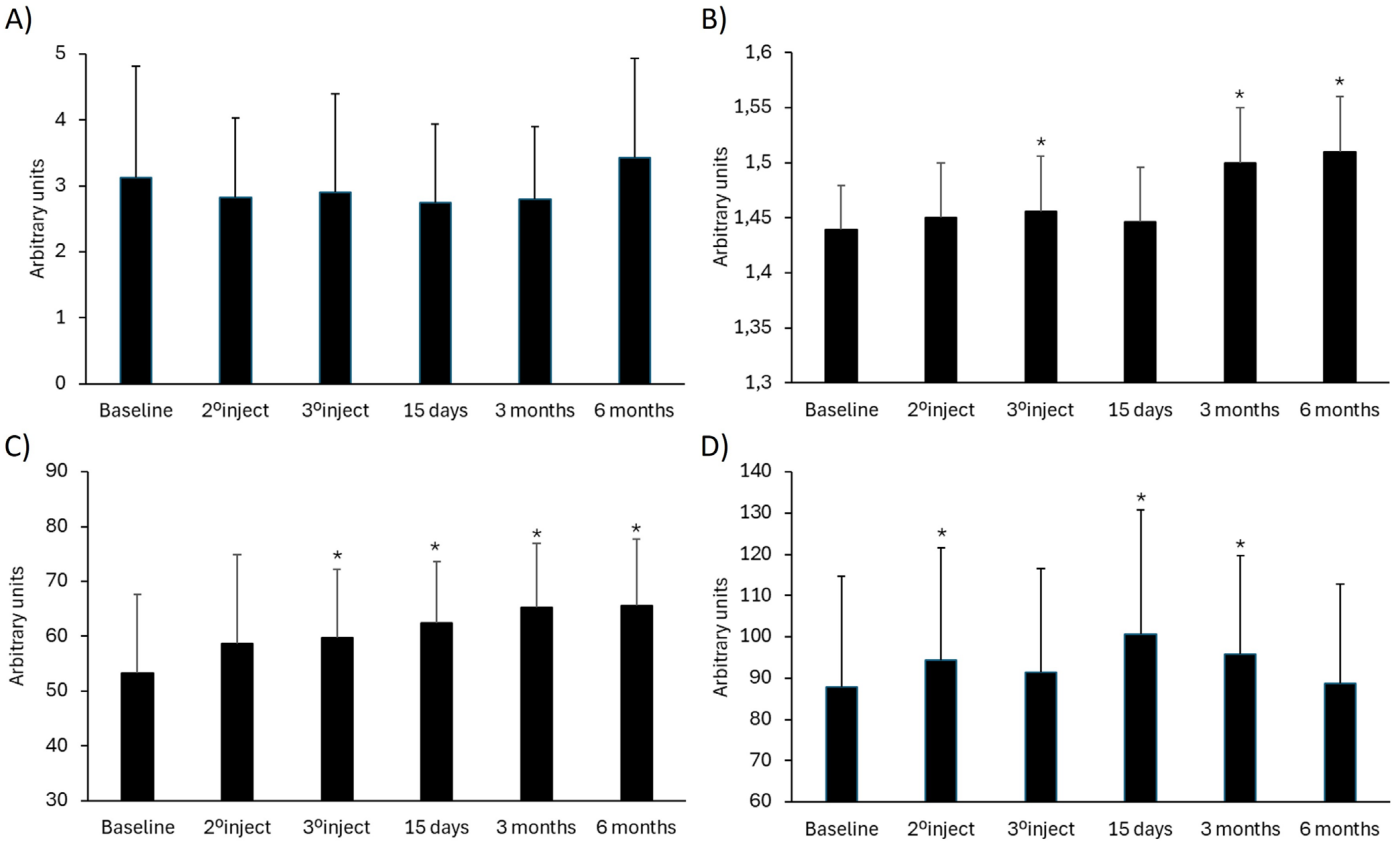
Global biometric analysis of the skin in response to BtHLINE throughout the study period. (A) Skin roughness. (B) Cutaneous uniformity. (C) Cutaneous viscoelasticity. (D) Water content. An increase in the biometric parameters is associated to a clinical improvement. 1°injection, 2°injection, 3°injection, 15 days, 3 months, and 6 months. *: Statistically significant differences compared to baseline (*p* ≤ 0.05). Data assessment comprises an analysis of all the included subjects.

**FIGURE 6:**
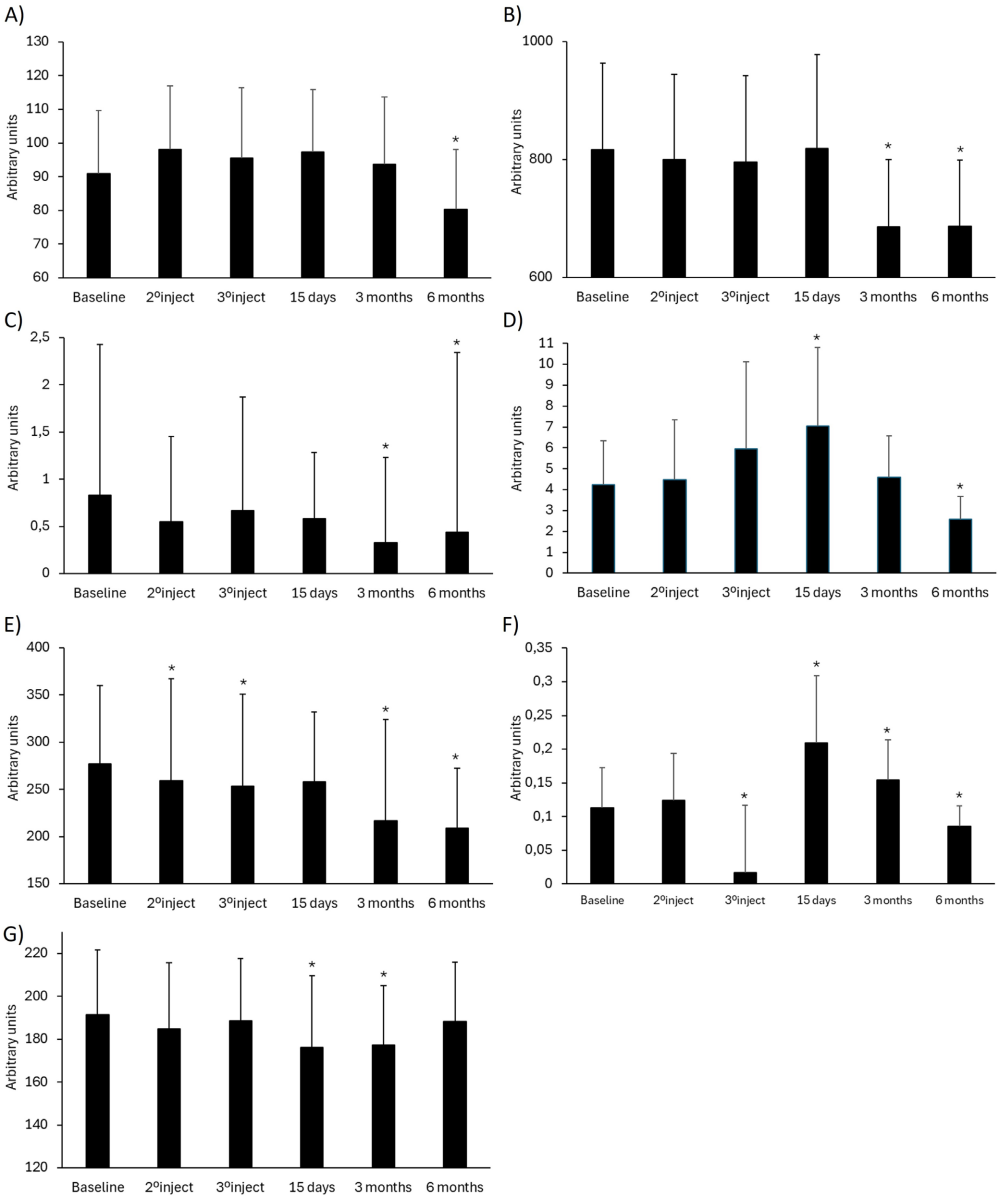
Global biometric analysis of the skin in response to BtHLINE throughout the study period. (A) Wrinkle depth. (B) Surface irregularities. (C) Scaling. (D) Firmness. (E) Smoothness. (F) Flexibility. (G) Hydration. A decrease in the biometric parameters is associated to a clinical improvement. 1°injection, 2°injection, 3°injection, 15 days, 3 months, and 6 months. *: Statistically significant differences compared to baseline (*p* ≤ 0.05). Data assessment comprises an analysis of all the included subjects.

**FIGURE 7:**
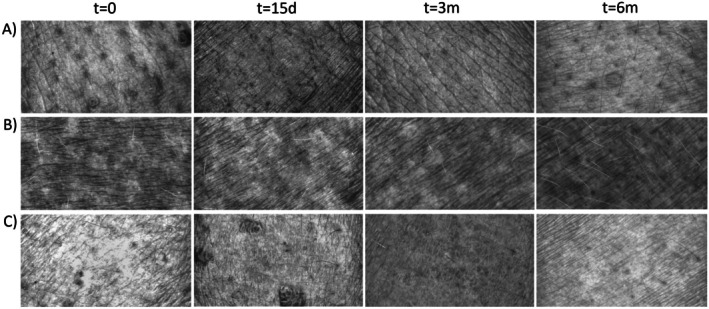
Representative gray scale images taken by biometric tools (Visioscan) for quantitative assessment. (A) Facial area. (B) Neck area. (C) Décolleté area.

The biometric analysis of the face revealed that generally, BtHLINE promoted a rapid and statistically significant improvement that was maintained during the follow‐up period. Representative Visioface assessment for wrinkle, pores, spots and color is depicted in Figure 4. In addition, topographic analyses using Visioscan, Cutometer and Moisturemap tools are shown in Figures 5 and 6. Specifically cutaneous uniformity, viscoelasticity and smoothness showed a steady improvement early after the first injection that was maintained up to 6 months. Other topographic parameters such as wrinkle depth, surface irregularities, scaling, firmness and flexibility reached a later statistical improvement after three to 6 months. Accordingly, the hydration and water content of the skin surface did also reveal an early improvement that persisted for 3 months. Figure 7 illustrates representative gray scale images taken by biometric tools for quantitative assessment.

## 4 Discussion

An ideal skinbooster should be biocompatible, adaptable to the patient's anatomy, non‐permanent but long lasting, reversible, with minimal side effects, easy to inject and cost‐effective. In addition, it should be a versatile tool for the treatment of different visible areas that are directly affected by age, such as the face, neck and décolleté [14]. HA is an endogenous polymer that has demonstrated its potential not only in the aesthetics field, but also in different medical areas such as traumatology, ophthalmology or dermatology [15, 16, 17]. Concerning this, researchers and manufacturers are developing technologies to select the ideal molecular weight and concentration of the HA and adjust its physiochemistry for proper physiological adaptation. Moreover, they are advancing on the optimization of the purity and origin of the raw materials while controlling final endotoxin levels [18].

BtHLINE is based on a high purity and high molecular weight HA (2.2 MDa) of biotechnological (non‐animal) origin (at an optimal concentration of 1.4% [14 mg/mL]). This hydrogel is a homogeneous and transparent solution without subvisible particles that is presented in a prefilled syringe of 1 mL which is terminally sterilized. BtHLINE has an adequate rheological profile that allows optimal injectability and an even distribution throughout cutaneous layers once intradermally injected. The product presents a physiological pH and osmolality, ensuring the biocompatibility of the product. Noteworthy the osmolality values meet the regulation established for ophthalmic viscosurgical devices (OVDs), which is more restricted than the common specifications that are usually ruled for general implants such as fillers or skinboosters. Similarly, the endotoxin levels of BtHLINE do also comply with a conservative value below 0.2 UE/mL which is directly related to a maximum prevention of hypersensitivity reactions. Hence, the technological optimization that stands behind the design and manufacturing process of BtHLINE could be related to the results reported in the present study.

In this clinical trial BtHLINE achieved an excellent clinical performance at the immediate (short), medium and long‐term (15 days, 3 months and 6 months respectively). It promoted an overall skin quality improvement with consistent results between the different scales and questionnaires that were fulfilled by both clinicians and volunteers during the follow up period. The clinical observations regarding radiance, hydration, firmness, fine lines and overall rejuvenation demonstrate a sustained effect of the product in all the treated areas that include the face, neck and décolleté. These findings are in accordance with other studies regarding cutaneous health and youthful appearance promotion after skinbooster use [19]. Moreover, no remarkable adverse events were reported being almost related to the injection procedure that disappeared within few days, which is common in this type of aesthetic procedures.

In addition, this study includes a deep biometric analysis of the cutaneous topography using validated and standardized tools in the skin care industry [20]. For most of the studied parameters, results revealed a statistically significant improvement after BtHLINE treatment. In fact, some parameters such as viscoelasticity, water content and hydration showed an immediate effect at day 15. Others like cutaneous uniformity, wrinkle depth, surface irregularities, scaling and smoothness showed a sustained improvement that peaked at the end of the follow‐up period (6 months). This could explain the patient satisfaction survey that demonstrated the highest responder rate at the end of the study. In contrast, other clinical trials have previously reported that the aesthetic efficacy of skinboosters tends to decrease before 6 months [21]. This is reasonable considering that, unlike dermal fillers, skinboosters present a limited HA concentration and absence of chemical crosslinking. Interestingly, BtHLINE demonstrated a consistent clinical performance even after 6 months. This may be attributed to the optimization of the inner structure of the hydrogel in terms of quality, purity, physiochemistry, molecular weight and rheological profile during the design and manufacturing process.

Unfortunately, the majority of scientific studies tend to focus on evaluating the efficacy and mechanism of action of dermal fillers rather than skinboosters. This may be due to the fact that dermal fillers often undergo significant physicochemical modifications that enhance their volumizing effect, such as cross‐linking reactions or polymer chain hybridization. As a result, there is an abundance of studies that analyze the clinical performance of dermal fillers based on their manufacturing technologies. In contrast, when it comes to linear HA skinboosters, the number and quality of studies are relatively lower, and the clinical evidence and commercial claims of these medical products are often based on known biological properties of HA rather than on objective clinical data. This is why the present study may help to unravel the specific mechanisms of action of this type of product, as it not only considers subjective perception scales but also quantitative biometric measurements.

Another noteworthy aspect of this clinical research is the analysis not only of the facial area—which is predominantly studied in scientific literature—but also of the neck and décolleté. It is well known that the morphology and structure of the skin can vary slightly depending on the anatomical location, which means that the response to the same treatment could differ. In this regard, this study provides valuable information about the mechanism of action of HA in different aesthetic areas that are common in daily rejuvenation procedures but lack robust scientific evidence.

Given the natural turnover of non‐crosslinked HA, the duration of skinbooster efficacy is rarely studied beyond 1–3 months. However, this study analyzes a new skinbooster that has been optimized in its manufacturing process by adjusting the origin, purity, molecular weight range, and concentration of HA polymer chains. Therefore, one of the novelties of this work was precisely to analyze results up to 6 months by observing persistent improvements in overall skin quality. Ultimately, and in synergy with previous studies, this clinical research may help to better understand the behavior of skinboosters and their impact in different anatomical areas over the long term.

Nevertheless, the current study presents some limitations such as the absence of a sub‐analysis based on the distribution of different ethnic groups, as well as a more nuanced interpretation of the results in relation to specific age cohorts and lifestyle factors, for example practicing sports or smoking status. Regarding this, future randomized and controlled clinical trials might corroborate the potential of BtHLINE as an effective hydrogel for boosting skin quality. Nevertheless, it should be highlighted that there are very few clinical studies that assess a skinbooster over different areas such as the face, neck and décolleté simultaneously and during prolonged time periods of up to 6 months. It is also remarkable the deep biometric assessment which has been carried out herein that ultimately provides quantitative data for an objective evaluation of the final clinical performance.

In conclusion, this study demonstrates that BtHLINE is safe and effective for the promotion of skin rejuvenation.

## Author Contributions

A.P.: Study design and manuscript writing. J.T.: Study design and manuscript writing. J.M.A.: Manuscript revision. L.T.: Clinical treatment and follow up. R.P.: Manuscript revision.

## Ethics Statement

The study protocol as well as the subject information sheet and informed consent form, were reviewed and approved on 30 March 2022 by the ethics committee of the University Hospital Principe de Asturias (code PIPS01/2022, Madrid, Spain) in accordance with the international ethical standards from the revised World Medical Association Declaration of Helsinki amended in 2013.

## Consent

Subjects provided written informed consent for photograph use.

## Conflicts of Interest

R.P. is the scientific director of i+Med. S. Coop., the company that commercializes BtHLINE. J.T., A.P. and J.M.A. are researchers at i+Med S. Coop. The other authors declare no conflicts of interest.

## Supporting information


**Table S1:** MAS Grading Scale.

## Data Availability

The data that support the findings of this study are available on request from the corresponding author. The data are not publicly available due to privacy or ethical restrictions.
