# Supplementary material for: Clinical and Biometric Assessment of a Hyaluronic Acid‐Based Skin Booster for Face, Neck and Décolleté Rejuvenation: A Prospective Study
Source: J Cosmet Dermatol. 2025 Nov 16;24(11):e70547. doi: 10.1111/jocd.70547 (PMC12620603; doi:10.1111/jocd.70547)
Supplement: Supplementary file 1 — Table S1: MAS Grading Scale. [file JOCD-24-e70547-s001.docx]

**Supplementary material**

Supplementary Table 1: MAS Grading Scale

|  | 0-very poor status | 1-poor status | 2-normal status | 3-good status | 4-very good status |
| --- | --- | --- | --- | --- | --- |
| Radiance | Absence of radiance | Slightly noticeable radiance | Moderately noticeable radiance | Noticeable radiance | Optimal level of radiance |
| Hydration | Absence of hydration | Slightly noticeable hydration | Moderately noticeable hydration | Noticeable hydration | Optimal level of hydration |
| Firmness | Absence of firmness | Slightly noticeable firmness | Moderately noticeable firmness | Noticeable firmness | Optimal level of firmness |
| Wrinkles/fine lines | Very pronounced and deep | Noticeably pronounced | Moderately pronounced | Slightly pronounced | Imperceptible |
| Overall rejuvenation | Very aged appearance | Slightly aged appearance | Appearance consistent with the biological age | Slightly rejuvenated appearance | Very rejuvenated appearance |
